# Visual backward masking: Modeling spatial and temporal aspects

**DOI:** 10.2478/v10053-008-0017-0

**Published:** 2008-07-15

**Authors:** Frouke Hermens, Udo Ernst

**Affiliations:** 1 Laboratory of Psychophysics, Brain Mind Institutem, École Polytechnique Fédérale de Lausanne (EPFL), Switzerland; 2 Group for Neural Theory, Département d’´Etudes Cognitives (DEC), École Normale, Supérieure (ENS), Paris, France

**Keywords:** visual backward masking

## Abstract

In modeling visual backward masking, the focus has been on temporal effects. More specifically, an explanation has been sought as to why strongest masking can occur when the mask is delayed with respect to the target. Although interesting effects of the spatial layout of the mask have been found, only a few attempts have been made to model these phenomena. Here, we elaborate a structurally simple model which employs lateral excitation and inhibition together with different neural time scales to explain many spatial and temporal aspects of backward masking. We argue that for better understanding of visual masking, it is vitally important to consider the interplay of spatial and temporal factors together in one single model.

## VISUAL BACKWARD MASKING: MODELING SPATIAL AND TEMPORAL ASPECTS

In visual backward masking, a target stimulus is followed by a mask, which impairs performance on the target. Although visual masking is often used as a tool in cognitive and behavioral sciences, its underlying mechanisms are still not well understood. The focus of masking research has been on understanding how it is possible that for some combinations of target and mask, a delay of the mask yields stronger masking than having the mask immediately follow the target. This phenomenon is known as ‘B-type masking’ or ‘U-shape masking,’ of which the latter refers to the shape of the curve linking stimulus onset asynchrony (SOA) between the target and the mask to performance. Explanations of B-type masking are either based on a single process (e.g. Anbar & Anbar, 1982; Bridgeman, 1978; Francis, 1997) or on a combination of two processes (e.g. Neumann & Scharlau, in press; Reeves, 1986). Most models which use a single process apply a mechanism which was termed ‘mask blocking’ by Francis (2000) . The basic idea of this mechanism is that a relatively strong target can block the mask’s signal at short SOAs, but fails to do so at intermediate SOAs due to the decaying trace of the target. The two process theories assume that the U-shape curve in B-type masking actually consists of two parts, both of which are monotonic. The two underlying processes might relate to the accounts of ‘integration’ and ‘interruption’ masking (Scheerer, 1973), or to ‘peripheral’ and ‘central’ processes (Turvey, 1973).

While the focus of visual backward masking has been on temporal aspects, the effects of the spatial layout of the target and the mask have received much less interest (but, see Cho & Francis, 2005; Francis & Cho, 2005; Hellige, Walsh, Lawrence, & Prasse, 1979; Kolers, 1962). If spatial aspects were investigated, they mainly involved low-level aspects, such as the spatial distance between the target and the mask, and the spatial frequencies of the stimuli. Recently, Herzog and colleagues (Herzog, Schmonsees, & Fahle, 2003a, b; Herzog & Fahle, 2002; Herzog & Koch, 2001; Herzog, Harms, Ernst, Eurich, Mahmud, & Fahle, 2003c; Herzog, Koch, & Fahle, 2001; Herzog, Fahle, & Koch, 2001) started to investigate the effects of the spatial layout of the mask systematically, while keeping the target (a vertical Vernier) constant. Even though the mask consisted of simple bar elements only, slight changes in the layout of these elements resulted in large differences in masking strengths. For example, adding two collinear lines to a grating mask strongly impaired performance on the Vernier target (Herzog, Schmonsees, & Fahle, 2003a).

Only a few modeling attempts have been made to explain spatial aspects of visual masking. The aspects that were modeled include the effect of the distance of the mask to the target (modeled by Breitmeyer & Ganz, 1976; Bridgeman, 1971; Francis, 1997), and the distribution of the mask’s contour (modeled by Francis, 1997). Several of the existing masking models (Anbar & Anbar, 1982; Di Lollo, Enns, & Rensink, 2000; Weisstein, 1968) are constructed in such a way that they cannot account for spatial aspects of the target and the mask.

Here, we describe a structurally simple model that can explain several spatial aspects of visual backward masking as well as temporal aspects. The model we use is inspired by the basic structures found in the visual cortex, with excitatory and inhibitory neurons driven by feed-forward input, and exchanging action potentials via recurrent horizontal interactions. We describe neural activity in terms of population firing rates, whose dynamics are similar to the classical Wilson-Cowan differential equations (Wilson & Cowan, 1973) for spatially extended populations. Here, we will present new simulations of the effects of a shift of the mask either in space or time, embedded in an overview of results earlier presented by Herzog et al. (Herzog, Ernst, Etzold, & Eurich, 2003; Herzog, Harms et al. 2003c).

## SETUP OF THE MODEL

**Figure 1.**
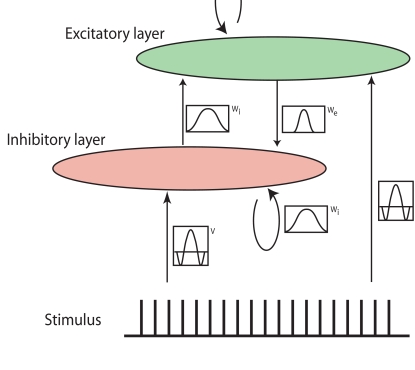
The general setup of the model. The input, which is coded as an array of ones and zeros is fed into an inhibitory and an excitatory layer via a Mexican-hat filter. The activation of these layers is updated over time.

The general structure of our model is illustrated in Figure 1. The input *I(x,t)* is filtered by a Mexican hat kernel and fed into an excitatory and an inhibitory layer. The activation of both layers is updated over time, where activation from both layers is mutually exchanged via the coupling kernels *W_e_* and *W_i_*. The activation dynamics of the model are determined by two coupled partial differential equations for the firing rates of neuronal populations, originally introduced by Wilson and Cowan (1973) . We modified the original equations in order to match more recent work (Ben-Yishai, Bar-Or, & Sompolinsky, 1995; Ernst, Pawelzik, Sahar-Pikielny, & Tsodyks, 2001) on the simulation of neural populations in the visual cortex, by dropping the shunting factors and using piecewise linear activation functions *h_e_* and *h_i_*, which do not saturate for high inputs,

(1) $$h_{e,i}(I)=\{\begin{array}{c} s_{e,i}I\;\text{for }I>0\\ 0\;\text{otherwise} \end{array}$$

with neuronal gain constants *s_e_* and *s_i_*. The activation in the excitatory (*A_e_*) and in the inhibitory layer (*A_i_*) is updated according to

(2) $$\tau_{e}\frac{\partial A_{e}(x,t)}{\partial t}=-A_{e}(x,t)+h_{e}\left[w_{ee}(A_{e}*W_{e})(x,t)-w_{ie}(A_{i}*W_{i})(x,t)+I(x,t)\right]$$

(3) $$\tau_{i}\frac{\partial A_{i}(x,t)}{\partial t}=-A_{i}(x,t)+h_{i}\left[w_{ei}(A_{e}*W_{e})(x,t)-w_{ii}(A_{i}*W_{i})(x,t)+I(x,t)\right]$$

In these equations, *τ_e_* and *τ_i_* denote time constants, and *w_ee_*, *w_ie_*, *w_ei_*, *w_ii_* are weighting coefficients for the interactions. *x* denotes the position of the neuronal population in the corresponding layer, and *t* denotes time. We assume an approximate retinotopical mapping of the visual input onto the cortical layer, such that *x* also describes position in the visual field.

Recurrent interaction between the layers is modeled by

(4) $$W_{e,i}(x-x')=\frac{1}{2\pi \sigma_{e,i}^{2}}\exp(-\frac{{\left(x-x'\right)}^{2}}{2\sigma_{e,i}^{2}}),$$

for excitatory and inhibitory interactions, respectively. The convolution, represented by *, describes the accumulation of synaptic inputs from other populations in the same or in a different layer. In the limit of large neuron numbers, it can be written as a spatial integral

(5) $$w_{ee}(A_{e}*W_{e})(x,t)=w_{ee}\int_{-\infty }^{+\infty }A_{e}(x',t)W_{e}(x-x')dx'$$

The feed-forward filtered input into both layers is computed by

(6) $$I(x,t)=(S*V)(x,t)=\int_{-\infty }^{+\infty }S(x',t)V(x-x')dx'$$

using an input kernel defined as a difference of Gaussians (DOG)

(7) $$V(x-x')=\frac{1}{\sqrt{2\pi }\sigma_{e}}\exp(-\frac{{\left(x-x'\right)}^{2}}{2\sigma_{e}^{2}})-\frac{1}{\sqrt{2\pi }\sigma_{i}}\exp(-\frac{{\left(x-x'\right)}^{2}}{2\sigma_{i}^{2}})$$

## SPATIAL ASPECTS

### Size of the grating

**Figure 2.**
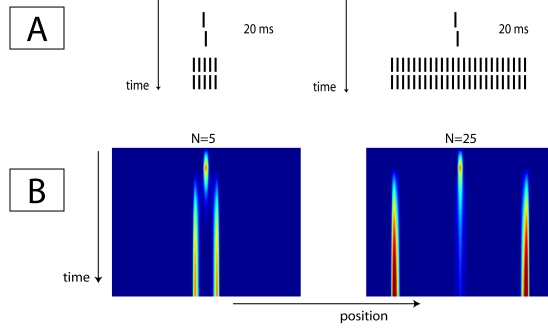
Stimulus sequence (A) and simulation results (B) of data presented by Herzog et al. (2001). A Vernier target was masked by a grating consisting of either five (left) or 25 elements (right). The model correctly predicts that the five-element grating masks the Vernier much more strongly than the 25-element grating.

**Figure 3.**
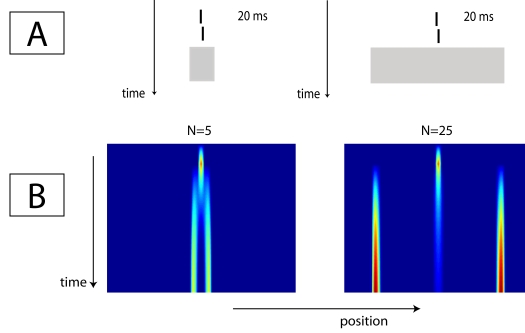
Stimulus sequence (A) and simulation results (B) of data presented by Herzog et al. (2003). A Vernier target was masked by a field of light of the size either of either five (left) or 25 elements (right). The model correctly predicts that the five-element size field masks the Vernier much more strongly than that of the size of a 25-element grating, as indicated by the longer Vernier trace for the 25-element grating in the center of the image of the network activation.

In their experiments, Herzog et al. (fig 3. Herzog, Fahle, & Koch, 2001) presented a Vernier target followed by a grating mask of a variable number of elements. Participants were asked to determine the offset direction (left or right) of the vertical Vernier. The mask consisted of an array of aligned vertical Verniers (as illustrated in Figure 2A). Masking was strongest when the grating consisted of 5 elements (about 58% correct decisions with a 20 ms Vernier duration), and weakest for gratings with more than 11 elements (about 91% correct).

First we will focus on an explanation of why the 5 elements yield stronger masking, while a larger mask (25 elements) yields weaker masking. Figure 2B shows the time evolution (vertical dimension) of the spatial activation in the excitatory layer (horizontal dimension). During the first 20 ms, the Vernier is presented, which results in a central activation of the layer. After these 20 ms, the Vernier input is ended and immediately the mask enters the system. For both gratings, this results in strong activation at the edges of the grating, strong inhibition in the surround of these edges, and suppression of all other activations. Since the edges of the five-element grating are much closer to the position where the Vernier was displayed, due to strong inhibition the remaining activation from the Vernier will decay faster than in the case of a 25-element grating.

To understand the consequences of these dynamics for perception, let us consider how activity in the model might be related to Vernier visibility. A common hypothesis is that the stronger an activation caused by a particular feature of a stimulus is, the better it can be detected by an observer of this activity. Consequently, the stronger the activation of the center column responding to visual input at the target’s position is, the better we expect the target to be visible, even it is blending over with the mask’s appearance, as in the typical reported percept of an observer in the 25-element condition. We therefore assume that the duration of the trace of activity associated with the center column, being above some threshold Θ, is monotonically related to visibility of the target element (linking hypothesis). It is therefore not necessary to model explicitly Vernier offset, as this feature of the target in the experiment is used only as a vehicle to quantify visibility. From elementary considerations in signal detection theory, it is obvious that the longer a noisy process is being observed, the better any estimation gets of some of its underlying parameters. The threshold in our case plays the role of an ad-hoc quantification of the neuronal background noise: only when activation increases beyond this noise level, may stimuli become visible. In order to quantify the linking hypothesis, one normally uses an experiment in which visibility or detection performance changes with some control parameter, and then fits a continuous function linking performance to a model variable. Once fixed, this function then allows prediction from the model how performance will be in other experimental conditions. While in a previous publication we employed this quantitative procedure, in this review article we only use qualitative measures, as e.g., predicting the peak performance in a specific condition, for evaluating the model’s performance.

### Uniform fields of light

In the previous paragraph, we saw that a grating of five elements masks a Vernier target much more strongly than a grating consisting of 25 elements. This finding was surprising, because the 25-element grating contains much more energy than the grating of 5 elements. The model suggested that the difference in masking strength could be explained by the distance of the nearest edge of the mask. If the distance to the edge of the grating is indeed what determines the masking strength, one would also expect a uniform field of light of the size of the five element grating to be a stronger mask than one of the size of the 25-element grating. Figure 3 shows that the model indeed predicts stronger masking for a small uniform field of light than for a large one. In the top part of this figure the sequence of stimuli is shown. The energy of the field of light was set such that the overall energy of the mask matched that of the corresponding grating mask. Figure 3B shows the activation over time for the two light masks. The pattern of results resembles that obtained for grating masks (Figure 2B). The small field of light suppresses the Vernier activation more strongly than the larger one.

Whether small fields of light mask more strongly than larger ones was experimentally investigated by Herzog, Harms et al. (2003c). Vernier offset discrimination thresholds indicated that the small light-field was indeed a stronger mask, although the difference in thresholds between the two mask sizes was not as large as for the grating masks. By using a function that linked network activation to thresholds (the ‘linking hypothesis’), Herzog, Ernst et al. (2003) showed that the model could accurately predict the observed thresholds.

### Irregularities in the mask

**Figure 4.**
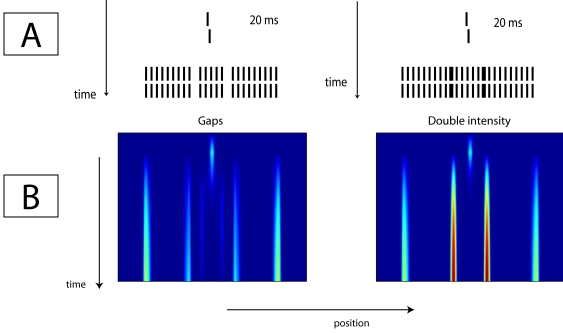
Stimulus sequences (A) and simulation results (B). A Vernier target was followed either by a grating with two gaps at offset positions +/-2 from the Vernier, or two elements of double luminance at these positions. Experimental data showed that both masks yield a strong increase in offset discrimination thresholds with respect to the standard grating. The simulations show that the model can well detect the irregularities in the mask, and explain how these irregularities result in an increase in masking strength. The irregularities are associated with strong network activation causing strong inhibition in their immediate surroundings that suppresses activation of the target, because the irregularities were close to the target.

Two findings suggest that breaking up the regularity of the mask increases its masking strength. Herzog et al. (fig. 4; 2001) introduced two gaps in the grating by removing two elements (illustrated in the left plot of Figure 4A), which resulted in a grating consisting of five central elements and two more distant groups of nine elements. The removal of the two grating elements strongly increased the strength of the mask. Similarly, Herzog et al. (fig. 7A; 2004) increased the luminance of the two elements at position offsets +2 and −2 from the Vernier, as illustrated in the right part of Figure 4A. Also, this slight change in mask layout resulted in a strong increase in the masking strength.

The simulation plots of Figure 4B show how we can understand the strong increase in masking strength by the introduction of the gaps or the double luminance elements into our model. The model is sensitive to irregularities in the grating, which yield high activations in the neuronal layers. As the activation induced by the gaps or by the elements with doubled luminance is close to the preceding Vernier activity, the decay of the Vernier activation will be faster, and thus predicted performance will be low.

The simulations with the mask with the two gaps show that not only the mask affects the target, but also the target affects the mask. The inner edges at the two gaps show weaker activation than the outer edges, which can be understood as resulting from stronger inhibition of the inner edges by the target than the outer edges. Said differently, the target forwardly masks the mask.

Masking is predicted to be slightly weaker for the mask with the two gaps than for the mask with double luminance lines. At this time, there is no experimental data to determine whether this prediction is correct. Thresholds were determined for both masks, however, with different observers with different amounts of training in the Vernier discrimination task. It would be interesting, though, to test this prediction in the future.

### Edge distance

**Figure 5.**
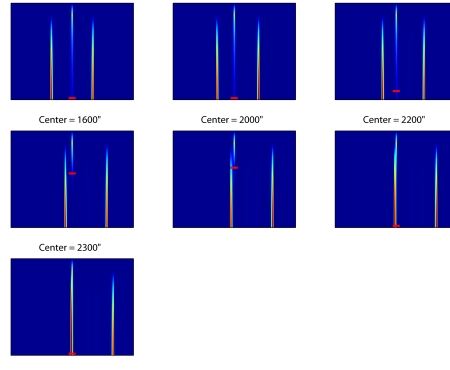
The activation in the excitatory population over time (vertical dimension) for different sizes of the shift of the center of the grating to the right. The small red horizontal bar indicates where the activity at the center drops below a certain value. The model predicts that when the grating’s edge approaches the Vernier, the Vernier’s trace is strongly reduced, implying much worse performance on the Vernier.

**Figure 6.**
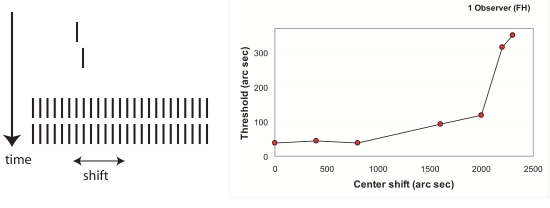
The sequence of Vernier and mask (left) and Vernier offset discrimination thresholds for observer FH (right) as a function of the size of the shift of the center of the grating mask. The data confirm the model’s prediction that a close edge yields strong inhibition of the Vernier’s signal, reflected in higher offset discrimination thresholds.

Previous simulations suggest that it is mainly the distance of the closest edge to the Vernier rather than the number of lines in the mask that determines the strength of the mask. This leads to the prediction that if the 25-element grating is shifted with respect to the location of the Vernier (as illustrated in the left part of Figure 6), masking strength will increase. This model prediction is illustrated in Figure 5, where the different subplots show the activation of the excitatory population across time (vertical dimension) for different sizes of the shift of the 25-element grating (the grating is shifted to the right of the center). The small red horizontal bar indicates where the activity at the center drops below a certain value. In the plot, a 0” shift indicates that both the Vernier and the grating were centered around the middle of the screen. A 400” shift indicates that the grating’s center was shifted 400” to the right, which means that the left edge of the grating is 400” closer to the Vernier compared to the standard situation. The model predicts that shifts up to 800” have little effect, while shifts larger than 1600” strongly affect the Vernier’s visibility. Note that merely looking at the moment the central activation drops below a certain value suggests a different pattern of results. This is because at some point the activation of the Vernier and that of the mask’s edge appear at the same spatial location. To avoid this confusion of activation, a different linking hypothesis might need to be used, or some spatial representation of the offset of the Vernier needs to be coded by the model.

Whether Vernier discrimination performance indeed decreases with an increasing shift of the mask was determined with one observer (author FH). This observer was presented with a sequence of a Vernier presented for 12 ms (the optimal duration for this observer), followed by a 25-element grating for 300 ms. The center of the grating was shifted from 0”, via 400”, 800”, 1600”, 2000”, 2200”’, to 2300” (edge close to the Vernier position), as is illustrated in the left part of Figure 6. For the rest, the experimental procedure was the same as in earlier demonstrations of the shine-through effect (e.g., Herzog, Harms et al., 2003c). The right part of Figure 6 shows the results. Thresholds start to rise at a shift of about 1600” (≈ ±8 elements offset), and reach a maximum for a shift of 2300” (≈ ±11.5 elements offset), where no threshold could be measured anymore.

The model was correct in predicting that thresholds increase with an increase in the shift of the mask. In addition, the model could well predict for which shift thresholds would strongly rise, which suggests that the model is correct in its assumption that the distance to the mask’s nearest edge determines the masking strength.

### Alternative explanations

Of the existing models of masking, only few are implemented in such a way that spatial information about the target and the mask can be coded (Bridgeman, 1978; Francis, 1997; Öğmen, 1993). Other computational models represent target and mask in single neurons (Anbar & Anbar, 1982; Di Lollo et al., 2000; Weisstein, 1968), an approach which does not allow spatial information to enter the model system.

Of the models that can code for spatial properties, only the model by Bridgeman can easily be implemented. The remaining two models (Francis, 1997; Öğmen, 1993) involve many stages and complex processing. For example, the model by Francis (1997) , which is based on the boundary contour system (Grossberg & Mingolla, 1985), consists of six layers with many complex interactions. To simulate these models, one probably needs the help of the authors to understand the full details in order to correctly implement the model. Moreover, these models often require simplifications of the model to be able to perform the simulations. Due to these restrictions, we will only present simulation results of Bridgeman’s model here.

The model by Bridgeman makes use of the Hartline-Ratliff equation that was originally developed to describe lateral inhibition in the Limulus eye. A network of 30 neurons is used to describe the effects of a visual mask. The firing rate of each neuron in the network changes over time depending on the excitatory sensory input and the inhibitory effect of neighboring neurons. To compare the network activations with the visibility of the target, the firing rates in the network are compared for a run in which only the target is presented, with one in which both the target and the mask are presented.

For the implementation of the Bridgeman model, we assumed a network of 500 neurons centered around the position where the Vernier was presented. In the original version of the model, 30 neurons were used, of which the first and the last neurons were linked to avoid edge effects. We choose a different approach: Since computers have become much faster, we could easily extend the number of neurons in the model, thereby avoiding edge effects (activation could not spread to the boundaries within the simulation time), while also avoiding neurons that were not close in retinotopic space affecting each other.

The background activation of the model was set to 50, additional activation of the target and mask was 22.5. The standard error of the Gaussian noise was assumed to be 0.1. The interaction parameters were the same as in earlier simulations by Bridgeman (1971, 1978). To initialize the network, 500 iterations were run in which only background activation was provided, before the stimuli were presented to the network. The target was presented for 2 time frames, the mask for the remaining 18 frames.

**Figure 7.**
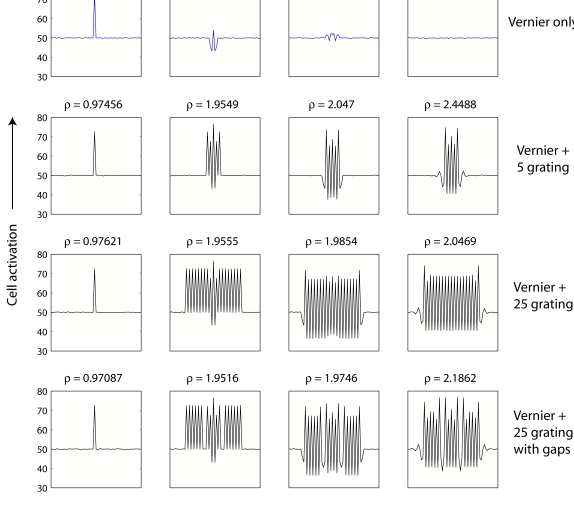
Cell activations in Bridgeman’s (1978) model for the conditions (1) Vernier only, (2) Vernier followed by a five-element grating, (3) Vernier followed by a 25-element grating, (4) Vernier followed by a 25-element grating with gaps. The value p in the subplot titles refers to the sum of the squared correlation over time between the activation for condition (1) and the respective condition. The higher the value of, the higher the predicted per-formance. The values indicate that the model fails to explain why a 5-element grating (2), and the 25-element grating with gaps (4) are much stronger masks than the 25-element grating (3).

Figure 7 shows the activation of the neurons at different points in time for different stimulus sequences. The top row shows the activation of the neurons after presentation of the Vernier only, the bottom three rows for a Vernier followed by one of three gratings (5-element grating, 25-element grating, 25-element grating with gaps, respectively).

The value in the subplots’ title (ρ) shows the sum over time of the squared correlation between the neuronal activation with the mask and that of the run without a mask. The sum is shown instead of the commonly used average, to make the outcome less dependent on the number of time steps in the simulation. If the model’s predictions agree with the data, we would expect to find a high value of ρ for the 25-element grating, and low values for the other two masks. This is not what is found: The value of ρ for the 25-element grating is, in fact, lower than that for the other two gratings, suggesting that Bridgeman’s model cannot account for the experimental findings.

## TEMPORAL ASPECTS

### Onset of context

**Figure 8.**
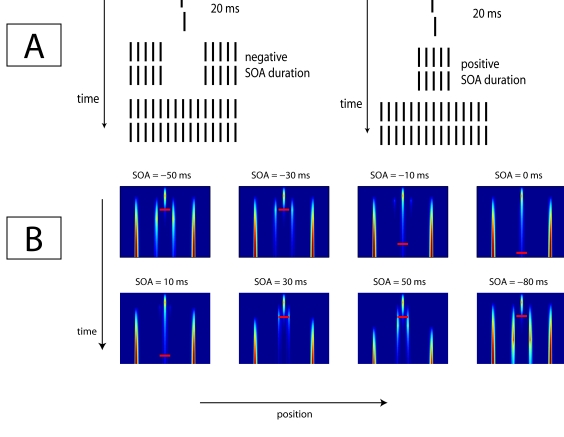
Stimulus sequence (A) and simulation results (B) of data presented by Herzog et al. (2001). The small red horizontal bars indicate where the activity of the trace drops below a particular threshold. A Vernier target was masked by a grating consisting of a five-element center and a 20-element surround, which were presented at different onset times. Once presented, the stimulus remained on the screen until 300 ms after target offset. The model correctly predicts that the target strength remains strongest for simultaneous onset of the mask’s center and surround.

 As discussed before, a grating of five elements is a stronger mask than one consisting of 25 elements (Herzog, Fahle, & Koch, 2001). Here, we will show simulation results in which the relative onset of the five central elements and the 20 surrounding elements of a 25-element mask was varied. Figure 8A shows the sequences used in the experiment by Herzog et al. (2001) . For negative SOAs, the 20 surrounding elements of the mask preceded the five central elements. For positive SOAs, the central five elements were presented before the surrounding 20 elements. At zero SOA, all 25 elements were presented simultaneously. In the experiment, Vernier offset discrimination thresholds were found to be minimal for an SOA equal to zero, and increased with SOA (either positive or negative). 

The Wilson-Cowan type model can explain why masking is weakest at zero SOA and increases with SOA. The activation plots that illustrate this are shown in Figure 8A. Each subplot shows the activation in the excitatory layer over time (vertical axis). The small red horizontal bars in the plots indicate where the activity of the trace drops below a certain threshold. During the first 20ms, the Vernier is presented to the network, followed by the sequence of mask parts. The duration over which the activation at the center of the population (where the Vernier was presented) survives is an indication of how well the Vernier will be perceived. The figure shows that the Vernier’s signal best survives for an SOA of zero, while the length of the Vernier’s trace decreases with increasing absolute SOA (either negative or positive).

Verbally, the explanation of the results can be phrased as follows. When the center and the surround are presented simultaneously, the network will consider the two parts as one object. The edges of this object are determined, and since they are far away from the Vernier target, they will hardly affect the signal of the Vernier. If the surround is presented earlier, the network will respond by detecting the edges of the two parts of the surround. Since the edges of these parts are much closer to the Vernier location, they will inhibit the Vernier more strongly. Similarly, if the center is presented before, its edges will be detected, and since also these edges are close to the Vernier, they will inhibit the Vernier’s signal. The trace of the mask in the population can change over time, as soon as other elements of the grating enter the network. This explains why early onset of the context elements results in a longer trace of the Vernier than late onset.

The model predictions were compared quantitatively with the experimental findings by Herzog et al. by applying a linking function converting the length of the suprathreshold trace of the Vernier into predicted thresholds (see model section). The model predictions closely matched the experimental results (Figure 6; Herzog, Ernst et al., 2003).

### Optimal masking at a non-zero SOA

**Figure 9.**
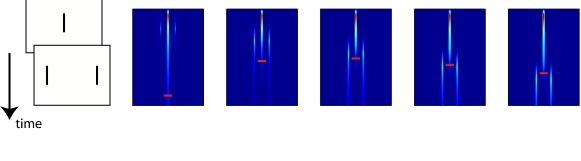
Stimulus sequence (left) and responses of the excitatory population (right) for which optimal masking at a non-zero SOA occurs. The small red horizontal bars indicate where the activity of the trace drops below a particular threshold. The Vernier’s trace is long for a zero SOA, then decreases in length for intermediate SOAs, and returns to full length again at long SOAs, indicating that masking is strongest at intermediate SOAs.

 In the introduction, we mentioned the relatively strong focus of the masking research community on explaining that masking can be strongest at a non-zero SOA (B-type masking). The work by Francis (2000) suggests that many models that apply a non-linearity (rectification) and decay can explain B-type masking. As our version of the Wilson-Cowan model contains both properties, we would expect that a combination of target and mask can be found for which the model shows strongest masking at a non-zero SOA. Figure 9 shows such a combination (left), together with the corresponding network responses (right). The small red horizontal bars indicate where the activity of the trace drops below a particular value. For short SOAs, the target’s trace is long. For intermediate SOAs, the length of the trace decreases, to increase again with longer SOAs. This pattern of trace lengths as a function of SOA suggests a U-shaped dependence of predicted performance on SOA. 

## GENERAL DISCUSSION

 In this paper, we have argued that it is important to study both spatial and temporal aspects of visual backward masking. Temporal aspects have been studied for a long time. Although some basic spatial aspects, such as the distance between target and mask, and their spatial frequencies have been studied in the past, it is only recently that spatial aspects have started to be investigated systematically. A similar trend can be seen for models of visual masking. Most earlier models (Anbar & Anbar, 1982; Weisstein, 1968) could only model temporal aspects of masking, simply because spatial aspects could not be coded by the models. An exception is the model by Bridgeman (1978) , which allows for a representation of stimuli in a spatial array. However, we showed that this model can not account for the difference in masking strength of the 25-element grating (weak masking), the five-element grating and the grating with two gaps (strong masking). Later models can represent the spatial layout of the stimuli, even in two dimensions (Francis, 1997; Öğmen, 1993). However, these models are so complex that a single simulation can take a standard computer days to perform (see the appendix of Francis, 1997), while at the same time preventing any analytical investigation of the relevant mechanisms. 

Here, we showed that a structurally simple cortical model with excitatory and inhibitory interactions can uncover putative mechanisms of several spatial and temporal aspects of masking. The model can explain why a grating of 5 aligned Vernier elements masks a Vernier target more strongly than one consisting of 25 elements. Similarly, it explains why a smaller uniform light-field masks more strongly than a large one. The model also correctly predicted that shifting the 25-element grating with respect to the Vernier target results in stronger masking. In addition to these spatial aspects of masking, the model could explain why a delayed onset of mask elements results in stronger masking, and how a non-monotonic relation between SOA and masking strength can be obtained.

The mechanisms which enable the model to work in the described way are easy to understand: The first stage of processing is a pure feed-forward filtering of the stimulus, realizing an edge enhancement (or detection of inhomogeneities) on the length scale of a typical double bar distance. The features of a stimulus pronounced by this procedure are then enhanced through a localized excitatory interaction, while two features within the distance of the length scale of the inhibitory interactions will compete for activation. A necessary condition hereby is that enhancement and competition are governed by two different time scales, a fast one for enhancement, and a slow one for competition. Through these time scales, features of mask and target are either superimposing or canceling each other. The most important aspect leading sometimes to counterintuitive effects is the strong recurrency in the interactions: even when a feature in the target, which leads to a pronounced activation in the network, has just been switched off, the excitatory interactions can sustain this activation for a prolonged period. During this period a competing, nearby feed-forward input of a mask has no chance to produce sufficient activation which in turn could suppress the target’s sustained activity. Only when this activity has decayed sufficiently, is the mask rendered effective. This mechanism in our model provides a putative neural basis for U-shaped masking curves.

By systematically comparing model output and experimental results, we can determine which aspects of masking can be explained with a simple mechanism, and which aspects need a more elaborate model. For example, the U-shaped dependence of performance on SOA for certain targets and masks can be explained with a single mechanism, and does not necessarily require two processes. However, Francis and Herzog (2004) showed that masking curves can intersect, even if the target and the task are kept constant, and just the mask is varied. This result poses strong restrictions on plausible models, suggesting that two or more neural processes underlie masking curves [as suggested by Reeves (1986) and Neumann and Scharlau (in press) ].

Computational models are also necessary to determine which conclusions can be drawn from data, as is illustrated by a recent contribution by Di Lollo and colleagues (2000) that received several comments. In their article, Di Lollo et al. suggested that no existing model could account for their data, and in particular for common onset masking, where the mask is onset at the same time as the target, but remains on the screen after target offset. They furthermore suggested that recurrent connections were needed to explain the results, instead of the feed-forward structure applied by existing models. The problem with their statements was that they did not check with simulations whether existing theories could already explain their data. Not much later, Francis & Hermens (2002) performed the necessary simulations and found out that common onset masking could easily be accounted for by existing models. Additional simulations then suggested which experiment would distinguish between the existing models and the newly proposed model by Di Lollo et al. (2000) . This experiment confirmed that the recurrent model by Di Lollo et al., in fact, outperformed all existing models (Francis & Cho, 2007).

The ultimate goal of modeling visual processing will be to construct a predictive model of the visual cortex. However, current computer capacities and also our current knowledge of the visual system do not allow this yet. Until the ultimate model of the brain can be constructed, we will have to work with much simpler models. The best strategy hereby is to tightly combine experimental and modeling studies to test upcoming theories of visual information processing, and to break down visual processing as far as possible into distinct modules which can under certain conditions be studied separately from each other. In such an integrative approach, we have demonstrated that a structurally simple cortical network can explain a quite extensive set of data in visual masking, which suggests that masking phenomena can be easily understood through the dynamics of network structures that are common to many areas found in the visual cortex.
